# An orally active plant Rubisco-derived peptide increases neuronal leptin responsiveness

**DOI:** 10.1038/s41598-022-12595-6

**Published:** 2022-05-21

**Authors:** Kentaro Kaneko, Yukihiro Takekuma, Tsuyoshi Goto, Kousaku Ohinata

**Affiliations:** 1 Division of Food Science and Biotechnology, Graduate School of Agriculture, Kyoto University, Gokasho, Uji, Kyoto 611-0011 Japan; 2 Department of Agricultural Chemistry, School of Agriculture, Meiji University, 1-1-1, Higashimita, Tama-ku, Kawasaki-shi, Kanagawa 214-8571 Japan

**Keywords:** Peptides, Obesity

## Abstract

Nutrient excess, such as the intake of a high-fat diet, reduces hypothalamic responses to exogenously administered leptin and induces dietary obesity; however, orally active components that attenuate neural leptin dysregulation have yet to be identified. We herein demonstrated that YHIEPV, derived from the pepsin-pancreatin digestion of the green leaf protein Rubisco, increased the leptin-induced phosphorylation of STAT3 in ex vivo hypothalamic slice cultures. We also showed that YHIEPV mitigated palmitic acid-induced decreases in leptin responsiveness. Furthermore, orally administered YHIEPV promoted leptin-induced reductions in body weight and food intake in obese mice. In addition, dietary-induced body weight gain was significantly less in mice orally or centrally administered YHIEPV daily than in saline-control mice. Cellular leptin sensitivity and the levels of proinflammatory-related factors, such as *IL1β* and *Socs-3*, in the hypothalamus of obese mice were also restored by YHIEPV. YHIEPV blocked cellular leptin resistance induced by forskolin, which activates Epac-Rap1 signaling, and reduced the level of the GTP-bound active form of Rap1 in the brains of obese mice. Collectively, the present results demonstrated that the orally active peptide YHIEPV derived from a major green leaf protein increased neural leptin responsiveness and reduced body weight gain in mice with dietary obesity.

## Introduction

Obesity is now a globally recognized health issue in children and adults. It is associated with serious co-morbidities, including type 2 diabetes, hypertension, cardiovascular disease, some cancers, and a reduced life expectancy. Weight loss is an effective strategy against a number of metabolic risk factors^1,2^.

Leptin is an adipocyte-derived peptide hormone that acts on the brain, particularly hypothalamic neurons. It has been shown to mediate energy homeostasis by inhibiting food intake, decreasing body weight, and enhancing systemic insulin sensitivity in lean humans and animals^3–6^. Circulating levels of leptin positively correlate with adiposity and body weight, and the central nervous system (CNS) receives peripheral energy information from leptin. However, an excess calorie intake, such as a high-fat diet (HFD), promotes the hypothalamic activation of multiple inflammatory and stress response pathways and induces the neural dysregulation of leptin. A decrease in the responsiveness of leptin impairs the central regulation of food intake and body weight, ultimately leading to obesity.

Recent studies suggested that G protein-coupled receptors (GPCRs), which activate cAMP-Epac-Rap1 signaling, impair leptin sensitivity in the hypothalamus^7–9^. cAMP accumulation (stimulated by forskolin)-induced cellular leptin resistance is mediated by Epac, a cAMP-regulated guanine nucleotide exchange factor for the small G-protein Rap1, but not by the classical cAMP effector, protein kinase A. Based on these data, we speculated that a GPCR ligand that activates Gs-cAMP and Rap1 signaling may also decrease leptin responsiveness. Through a combination of pharmacological, mouse genetic, and brain explant studies, we demonstrated that the gut-derived incretin hormone glucose-dependent insulinotropic polypeptide, also known as gastric inhibitory polypeptide (GIP), acted on the hypothalamic metabolic circuitry to drive leptin and insulin resistance as well as obesity by activating Epac-Rap1 signaling^10,11^. Based on these findings, we hypothesized that peripherally-derived exogenous GPCR ligands, such as food-derived bioactive peptides, promote leptin sensitivity in the hypothalamus by inhibiting Epac-Rap1 signaling.

Recent studies reported that dietary proteins are as a source of bioactive peptides^12–14^. A number of bioactive peptides derived from the enzymatic digestion of food proteins in the gastrointestinal tract have been identified. These peptides have specific amino acid sequences and exhibit various physiological activities beyond their nutritional value through the digestive, endocrine, cardiovascular, immune, and central and peripheral nervous systems. Ribulose-1,5-bisphosphate carboxylase/oxygenase (Rubisco), a key enzyme for photosynthetic carbon dioxide fixation and photorespiration in plants and other organisms, is the most abundant protein on the Earth because it accounts for approximately 30–50% soluble protein in the green leaves of plants^15^. Therefore, we selected Rubisco as an attractive and sustainable source of bioactive peptides.

To identify food-derived bioactive peptides that increase neural leptin responsiveness, we adapted a screening method using ex vivo organotypic hypothalamus slice cultures with previously identified Rubisco-derived bioactive peptides. We found that a treatment with Tyr-His-Ile-Glu-Pro-Val (YHIEPV), derived from the enzymatic digestion of Rubisco with pepsin and pancreatin, mimicking enzymatic conditions in the gastrointestinal tract, increased the leptin-induced phosphorylation of STAT3, a marker of activated leptin signaling. Therefore, the present study investigated the activities of and mechanisms by which YHIEPV increases leptin sensitivity to exert its anti-obesity effects.

## Materials and methods

### Materials

YHIEPV was synthesized by the F-moc strategy and purified by reversed-phase high-performance liquid chromatography (HPLC). Forskolin was obtained from Tocris Bioscience. Recombinant murine leptin was obtained from PEPROTECH. Palmitic acid was obtained from Tokyo Chemical Industry.

### Animals

Mice were used for all experiments. Male C57BL/6 and ddY mice were both obtained from Japan SLC (Shizuoka, Japan). All mice were maintained on a 12-h light/dark cycle (lights on 7 a.m.–7 p.m.) in a temperature-controlled environment at 22–24 °C with ad libitum access to water and a normal diet (CE-2, CLEA Japan) or HFD (60% kcal fat; Research diet, D12492). In the present study, ddY mice were used to examine the effects of an oral YHIEPV treatment, while C57BL/6 mice were used for the central YHIEPV treatment and in the hypothalamic signaling analysis. All experimental procedures were performed in accordance with the ARRIVE guidelines. The care of all animals and procedures were approved by the Kyoto University Animal Committee and Meiji University Animal Committee. Animal experimentation guidelines were followed.

### Cannula implantation and treatments

Mice were anesthetized with isoflurane and positioned in a stereotaxic frame. The skull was exposed and a 26-gauge single stainless steel guide cannula (C315GS-5-SPC, Plastics One, Roanoke, VA, USA) was implanted into the lateral ventricle (− 0.45 mm from the bregma, ± 0.9 mm lateral, − 2.5 mm from the skull) as previously described^8^. The cannula was secured to the skull with screws and dental cement. After i.c.v. cannulation, mice were housed singly and given at least 1 week to recover. On experimental days, mice were infused with 1 μL of the following solutions: vehicle (saline), leptin (0.5 μg/mouse) or YHIEPV (1 μg/mouse). At the end of the experiment, the placement of the guide cannula was verified histologically.

### Effects of the daily oral administration of YHIEPV

Male ddY mice were singly housed and acclimatized for 1 week prior to experiments. Body weight-matched 8-week-old mice were fed HFD for 2 months. Control (saline) or YHIEPV (0.3 mg/kg/day) was orally administered once a day. The dosage of YHIEPV was selected based on a previous study^16^. The administration of peptide solution was performed by oral gavage. Body weights and food intake were measured daily. At the end of the experiment, after 3 h of fasting with free access to water, blood samples were collected from the orbital sinus of mice under deep anesthesia with isoflurane into a tube rinsed with EDTA and then centrifuged (3,500 rpm, 4 °C, 10 min) to obtain plasma samples, which were stored at − 80 °C for later analyses.

### Leptin sensitivity test

Male ddY mice were fed HFD. After 4 weeks of HFD feeding, an intracerebroventricular (i.c.v.) cannula was implanted and mice were allowed 1 week to recover. YHIEPV (0.3 mg/kg/day) was administered at 5 p.m. for 3 days, and mice were then treated with leptin (0.5 µg/mouse) or saline once a day at 5 p.m. for 3 consecutive days. Food intake and body weights were measured daily.

### Organotypic hypothalamus slice culture

The organotypic slice culture was performed as previously described^17^. Hypothalamic slices were obtained using a previously reported method with slight modifications^8^. C57BL/6 pups at 8–11 days old were decapitated and the brains were quickly removed. Hypothalamic tissues were sectioned at a depth of 250 μm on a vibratome (Leica VT1200S) in chilled Gey’s balanced salt solution enriched with glucose (0.5%) and KCl (30 mM). Coronal slices containing the arcuate nucleus (ARC) were then placed on Millicell-CM filters (Millipore, pore size 0.4 μm, diameter 30 mm) and maintained at an air-medium interface in minimum essential media supplemented with heat-inactivated horse serum (25%), glucose (32 mM), and GlutaMAX (2 mM). Cultures were typically maintained for 8–10 days in standard medium, which was replaced 2 or 3 times a week. Healthy slices typically showed a slight reduction in the thickness of the hypothalamus after 10 days of incubation, while marked decreases in unhealthy slices, which mostly lost the hypothalamic structure. After 10 days, the selected healthy slices were then randomly divided into experimental and control groups.

### Immunohistochemistry

Immunohistochemistry was performed as previously described^8^. Under deep anesthesia, male ddY mice were intracardially perfused with saline and 4% paraformaldehyde. The brains were removed, post-fixed in 4% paraformaldehyde, infiltrated with 20% sucrose, and cut into 30-μm-thick slices. Sections were rinsed 6 times for 5 min each in PBS and then placed in 0.3% hydrogen peroxide in 0.25% Triton X-100 in PBS (PBT) for 30 min. Sections were then incubated for 48–72 h with phosphorylated STAT3 (pSTAT3) antibodies (1:3000, Cell Signaling Technology, 9131) in 3% normal donkey serum with PBT and 0.02% sodium azide. Sections were reacted with a biotinylated secondary antibody against rabbit IgG (1:1000, Jackson ImmunoResearch Labs, West Grove, PA) followed by the avidin–biotin–peroxidase complex (ABC) kit (1:1000, Vectastain Elite ABC kit; Vector Labs, Burlingame, CA). Immunoreactivities were visualized by an incubation with 3,3′-diaminobenzidine (DAB, Sigma, St. Louis, MO). After dehydration through a graded series of ethanol, slides were immersed in xylene and coverslipped. Images were analyzed using a brightfield Olympus microscope. Organotypic hypothalamic slices were cut from the membrane, rinsed 3 times for 10 min each in PBS at pH 7.4, and then placed for 20 min in 1% hydrogen peroxide and 1% sodium hydroxide in PBS to quench endogenous peroxidase activity. Following a series of washes with PBS, slices were incubated at 4 °C for 48–72 h in pSTAT3 antibodies (1:3000) in 3% normal donkey serum (Jackson ImmunoResearch Labs) with PBT and 0.02% sodium azide. After washing in PBS, slices were incubated with a biotinylated donkey anti-rabbit antibody (1:1000) in 3% donkey serum in PBT at room temperature for 1 h. Tissues were rinsed in PBS and incubated in ABC (1:500) for 1 h. Slices were washed in PBS and then reacted with DAB. Slices were then rinsed in PBS and mounted on slides using Vectashield (Vector Labs). To measure the pSTAT3 signal in the ARC, the uneven background was eliminated with Adobe Photoshop and the intensity of DAB staining was measured with NIH ImageJ.

### Quantitative RT-PCR

Hypothalamic samples were collected from C57BL/6 mice and total RNA was extracted using the RNeasy Mini Kit (QIA-GEN, Hilden, Germany). cDNA was generated by the Takara PrimeScript^®^ RT Master Mix (Takara, Osaka, Japan). Regarding quantitative real-time PCR, we amplified cDNA using QuantStudio 1 (Thermo Fisher SCIENTIFIC, Inc., Waltham, MA, USA) with the THUNDERBIRD^®^ qPCR Mix (Toyobo Co., Osaka, Japan) and each primer set specific for mouse *cyclophilin*, *suppressor of cytokine signaling-3 (SOCS-3)*, *protein tyrosine phosphatase 1B (PTP1B)*, *T-cell protein tyrosine phosphatase* (*TCPTP)*, *IL6*, *IL1β*, or *TNFa* according to the manufacturer’s instructions as previously described^8^. Reactions were cycled 40 times with denaturation at 95 °C for 15 s and with annealing and elongation at 60 °C for 60 s. Normalized mRNA levels were expressed in arbitrary units obtained by dividing averaged efficiency-corrected values for sample mRNA expression by that for *cyclophilin* mRNA expression for each sample.

### Detection of active-Rap1 by the Rap1 pull-down assay

A Rap1 pull-down assay was performed using the active Rap1 pull-down and detection kit (Thermo Fisher Scientific) according to the manufacturer’s recommendations as previously described^8^. After brain samples were dissected from C57BL/6 mice, samples were snap-frozen and subsequently stored at − 80 °C. Proteins were extracted by the provided lysis/wash buffer with a protease cocktail inhibitor, and lysates were then centrifuged at 16,000 × *g* at 4 °C for 15 min. Protein concentrations were measured using the BCA protein assay (Pierce, 23225) with BSA as the standard. Equal amounts of protein were subjected to the affinity precipitation of guanosine triphosphate (GTP)-Rap1 using the active Rap1 pull-down and detection kit. The amount of Rap1 was measured by performing Western blotting with the antibody provided (1:1000). Equal amounts of samples were separated by SDS-PAGE and transferred to a nitrocellulose membrane by electroblotting. After an incubation with the primary antibodies at 4 °C for 24 h, the membranes were incubated with the anti-rabbit IgG secondary antibody at room temperature for 1 h. Fluo Chem 8800 (Alpha Innotech) was used to measure the intensity of active-Rap1. To assess total Rap1 levels, the cell extract was directly subjected to Western blotting without the pull down of proteins.

### Food intake experiment

Male ddY mice were fed HFD. After 4 weeks of HFD feeding, an intracerebroventricular (i.c.v.) cannula was implanted and mice were allowed 1 week to recover and individually housed with free access to water and food pellets. Mice were received YHIEPV (0.3 mg/kg, p.o.) or vehicle (saline) treatment, followed by HS024 (0.1 nmol/mouse, i.c.v.). Food intake were measured after 4 h of treatment.

### Statistical analysis

Data are presented as the mean ± SEM. Statistical analyses were performed using GraphPad Prism 9 for the two-tailed unpaired Student’s *t*-test, or a one- or two-way ANOVA followed by Tukey’s, Bonferroni, or Sidak’s post hoc tests. P < 0.05 was considered to be significant.

### Study approval

All procedures to maintain and use mice followed protocols reviewed and approved by the Animal Research Committee of Kyoto University (Kyoto, Japan) and Meiji University (Kanagawa, Japan).

## Results

### YHIEPV increases cellular leptin sensitivity

**Figure 1:**
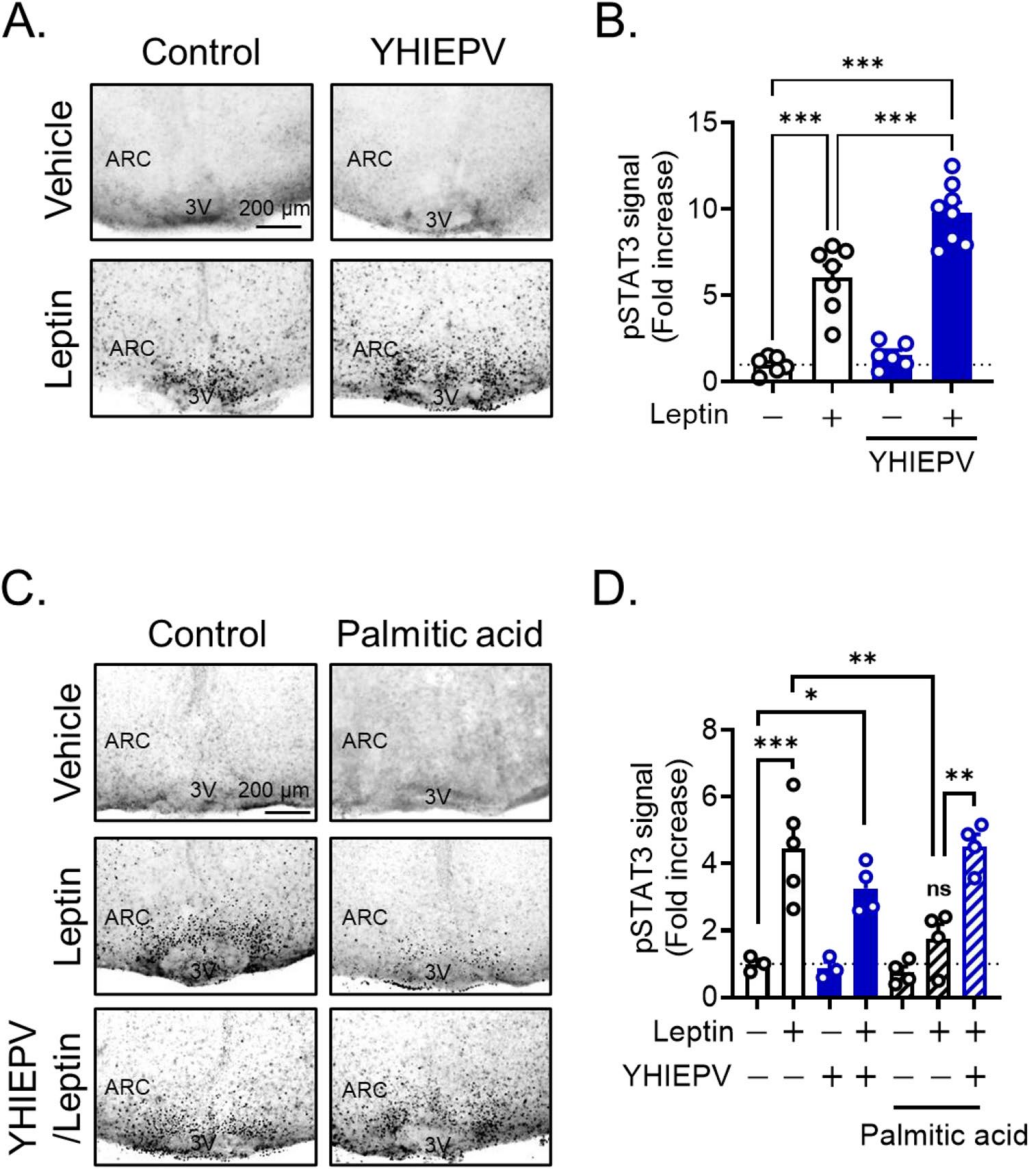
YHIEPV promotes intracellular leptin sensitivity. (**A**, **B**) Effects of YHIEPV on cellular leptin sensitivity in organotypic hypothalamus slices. Hypothalamic slices were made from C57BL/6 pups. Slices were incubated with YHIEPV (100 μM, 24 h) and then stimulated with leptin (30 nM, 60 min). (**A**) Representative immunohistochemistry images for pSTAT3 in fixed slices. Scale bar: 200 μm. (**B**) Quantification of hypothalamic pSTAT3 (n = 6–8/group) in organotypic hypothalamus slices. (**C**, **D**) Effects of YHIEPV on palmitic acid-induced cellular leptin resistance in organotypic hypothalamus slices. Slices were incubated with palmitic acid (30 μM) in the presence or absence of YHIEPV (100 μM) for 6 h and then stimulated with leptin (60 nM, 60 min). (**C**) A leptin-induced pSTAT3 image is shown. Scale bar, 200 μm. (**D**) Quantification of hypothalamic pSTAT3 (n = 4–5/group) in organotypic hypothalamus slices. *p < 0.05, **p < 0.01, ***p < 0.001 for a one-way ANOVA followed by Tukey’s multiple comparisons tests in (C and D). All error bars are SEM.

We initially investigated whether YHIEPV positively regulated hypothalamic leptin sensitivity using organotypic hypothalamic slice cultures. The organotypic hypothalamus slice cultures could reproduce a part of leptin signaling, such as leptin-dependent pSTAT3, a critical mediator for leptin’s metabolic effects and a marker of activated leptin signaling^18–21^. Cellular leptin sensitivity may be evaluated by leptin-induced pSTAT3 levels. In this study, we have thus used the organotypic hypothalamus slice cultures as an in vitro model of cellular leptin signaling. pSTAT3 was observed in control slices stimulated with leptin (30 nM), while the treatment of organotypic hypothalamus slices with YHIEPV (100 μM, 24 h) significantly increased leptin-induced pSTAT3 levels (Fig. 1A,B). This result suggested that YHIEPV increased leptin sensitivity.

HFD is rich in saturated fatty acids, such as palmitic acid, which has been shown to induce central leptin dysregulation^22–24^. Therefore, we modeled cellular leptin resistance by treating organotypic hypothalamus slices with palmitic acid to induce cellular leptin resistance. Leptin-induced pSTAT3 levels were significantly reduced by palmitic acid (30 μM, 6 h) (Fig. 1C,D). A pretreatment with YHIEPV (100 μM, 6 h) abolished palmitic acid-induced cellular leptin resistance in ex vivo slices (Fig. 1C,D). This result suggested that the YHIEPV treatment increased leptin responsiveness and prevented HFD-induced reductions in leptin sensitivity.

### Orally administered YHIEPV increases hypothalamic leptin sensitivity in HFD-induced obese mice

**Figure 2:**
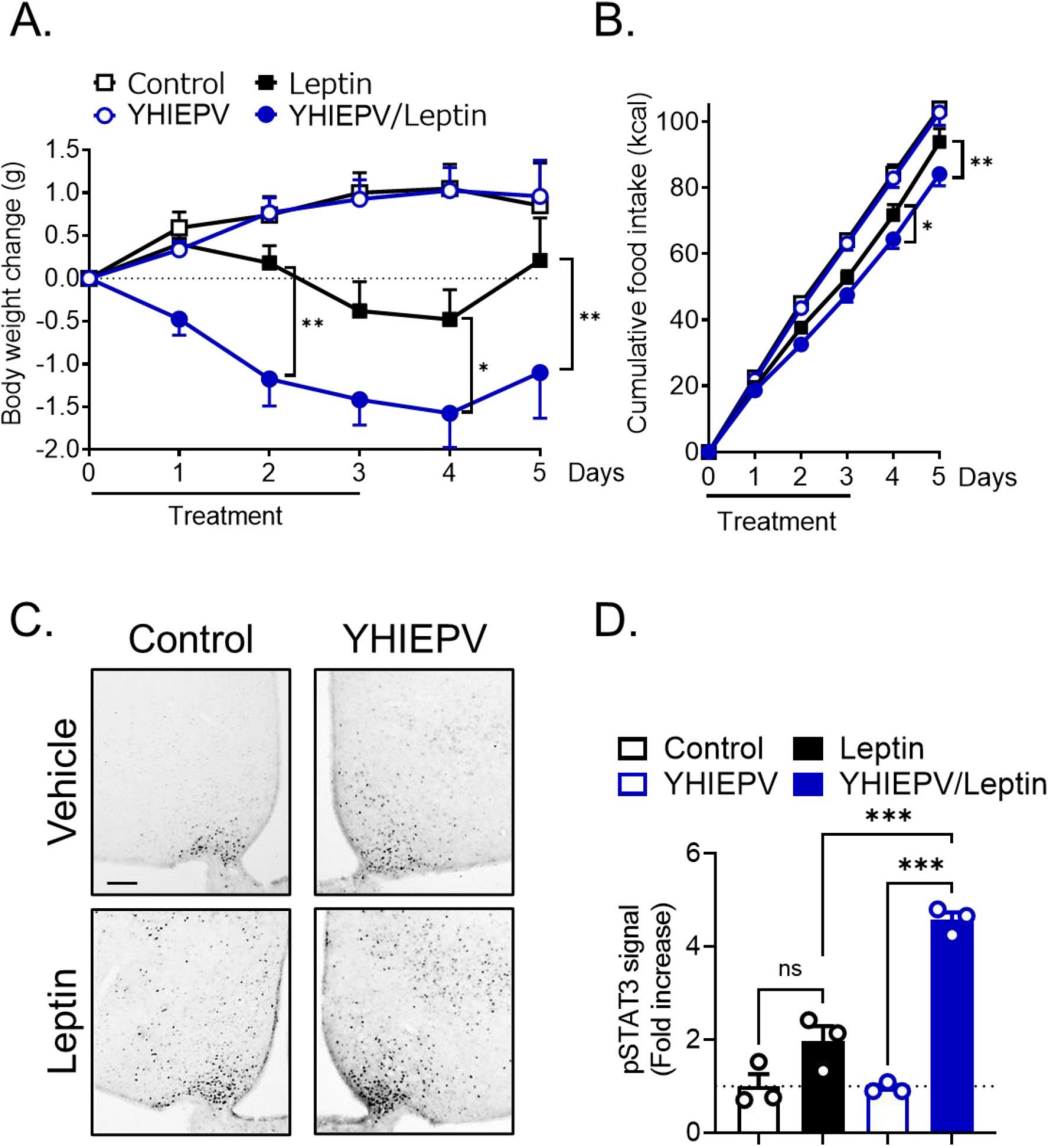
Orally administered YHIEPV increases leptin sensitivity in HFD-fed mice. (**A**, **B**) The oral administration of YHIEPV increased leptin-induced reductions in body weight (**A**) and food intake (**B**). YHIEPV oral injections (0.3 mg/kg) were started 3 days before leptin injections. Leptin (0.5 μg) or vehicle was i.c.v. infused with or without YHIEPV (0.3 mg/kg, p.o.) to HFD-fed ddY mice (HFD for 5 weeks, control: 48.30 ± 1.4 g, YHIEPV: 47.29 ± 1.8 g, Leptin: 49.68 ± 1.8 g, YHIEPV/Leptin: 49.32 ± 2.1 g, on the leptin experimental day) (n = 10–12/group). (**C**, **D**) Leptin (0.5 μg, i.c.v., 60 min) was administered to the indicated ddY mice (n = 3/group). YHIEPV was orally injected for 3 days. (**C**) Representative immunohistochemistry images for pSTAT3. Scale bar, 100 μm. (**D**) Quantification of immunohistochemistry. *p < 0.05, **p < 0.01, ***p < 0.001 for a two-way ANOVA followed by Tukey’s multiple comparisons tests in (**A**) and (**B**) or a one-way ANOVA followed by Tukey’s multiple comparison test in (**D**). All error bars are SEM.

We examined whether orally administered YHIEPV increased leptin responsiveness in diet-induced obese mice. Mice were fed HFD for 5 weeks. Using age and body weight-matched groups, we assessed the anorectic response to leptin following its central administration. YHIEPV or saline was orally administered for 3 days before the i.c.v. injection of leptin. Mice orally administered YHIEPV gained significantly less body weight and had a lower cumulative food intake during the central leptin injection than control obese mice (Fig. 2A,B). This result suggested that orally administered YHIEPV increased leptin sensitivity in the hypothalamus. To confirm this hypothesis, we performed an immunohistochemical analysis. The co-administration of YHIEPV (0.3 mg/kg, p.o., 3 days) and leptin (0.5 µg, i.c.v.) markedly sensitized leptin-responsive neurons in the hypothalamus, as demonstrated by the restoration of leptin-induced pSTAT3 levels; however, this sensitization was absent in control mice under HFD conditions (Fig. 2C,D). Therefore, the oral administration of YHIEPV increased hypothalamic cellular leptin signaling under HFD conditions.

### YHIEPV protects against HFD-induced obesity

**Figure 3:**
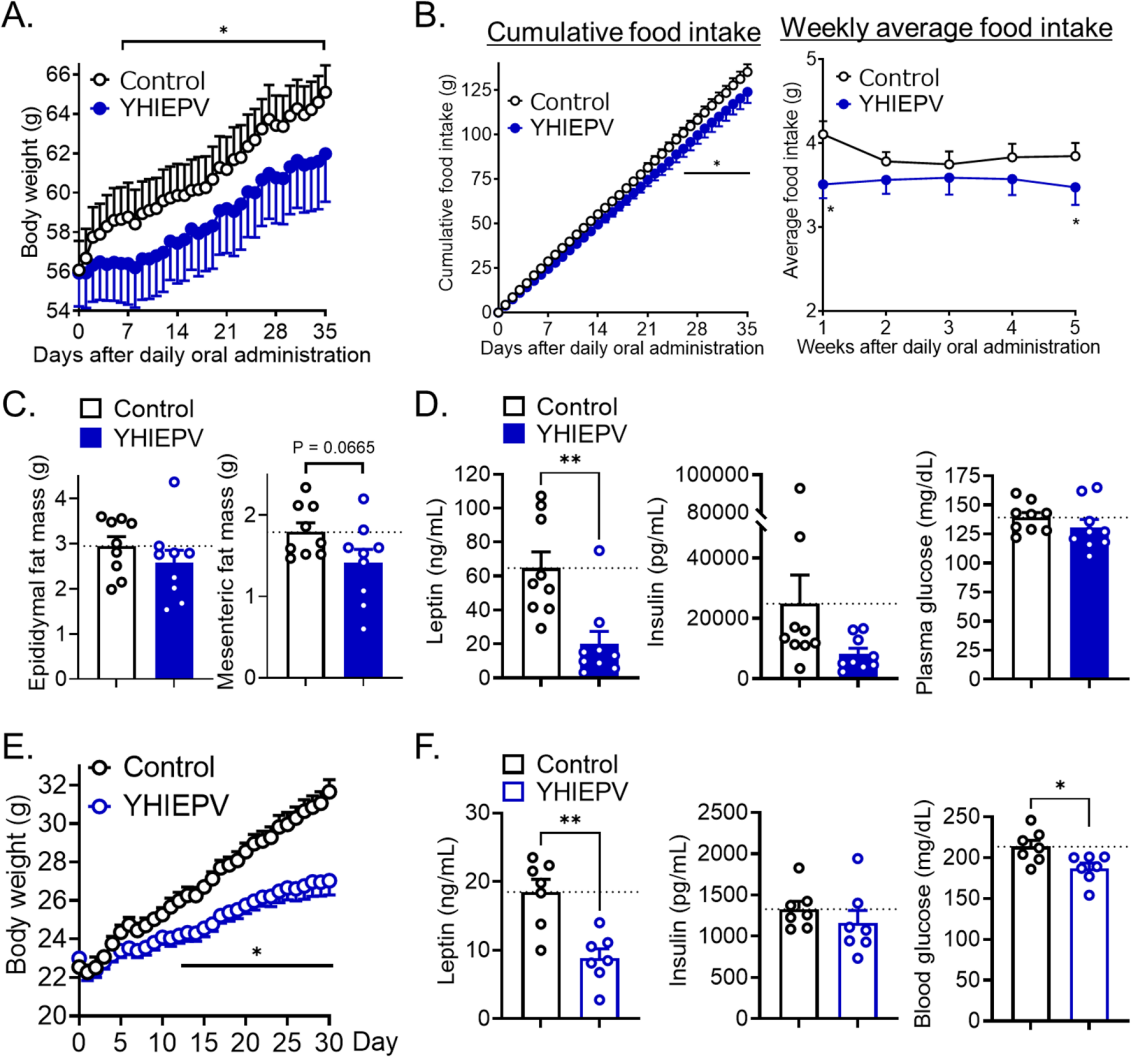
YHIEPV protects against dietary obesity. (**A**–**D**) Effects of YHIEPV on body weight and food intake in HFD-induced obese ddY mice. Body weight changes (**A**) and cumulative food intake or weekly average food intake (**B**). HFD-fed obese ddY mice (HFD for 2 months, n = 9/group) were orally administered YHIEPV (0.3 mg/kg for 35 days) once a day. Epididymal and mesenteric fat masses (**C**) and plasma leptin, insulin, and glucose levels (**D**) were measured on day 35. Mice were fasted for 3 h (from 8 to 11AM) before the collection of tissue and blood samples. (**E**, **F**) YHIEPV was centrally infused (1 μg, every day) into C57BL/6 mice (n = 7). HFD was initiated on day 0. Body weight (**E**) was measured daily. Plasma levels of leptin and insulin and blood glucose levels (**F**) were measured on day 30. Mice were fasted for 3 h (from 8 to 11AM) before the collection of blood samples. *p < 0.05, **p < 0.01, ***p < 0.001 for a two-way ANOVA followed by Bonferroni multiple comparisons tests in (**A**–**C**) or *t*-tests in (**D**) and (**F**). All error bars are SEM.

We examined the effects of YHIEPV on diet-induced obesity and associated metabolic disturbances. We employed a long-term administration protocol for HFD-induced obese mice. Body weight gain, cumulative food intake, and weekly average food intake were significantly lower in HFD-induced obese mice orally administered YHIEPV daily (0.3 mg/kg/day) than in saline-control mice (Fig. 3A,B). Although mesenteric and epididymal fat masses were slightly reduced (Fig. 3C) and plasma insulin and adiponectin levels remained unchanged (Fig. 3D and Fig. S1), plasma leptin levels were markedly reduced by YHIEPV (Fig. 3D). Since leptin is produced by adipose tissue and plasma leptin levels correlate with percent body fat, these results suggested that decreased plasma leptin levels correlated with enhanced leptin sensitivity and a slightly reduced fat mass in YHIEPV-treated mice. To further confirm the body weight phenotype of the YHIEPV treatment, we performed a daily stereotaxic injection of YHIEPV into the lateral ventricle. Mice were fed HFD immediately after the first i.c.v. injection of YHIEPV to assess whether YHIEPV protects against diet-induced obesity. In comparisons with control mice, the central administration of YHIEPV daily markedly reduced body weight gain and blood glucose levels (Fig. 3E,F). Therefore, green leaf Rubisco-derived YHIEPV exerted the anti-obesity effects.

### Rap1 signaling in the CNS is involved in YHIEPV-induced increases in leptin responsiveness

We investigated the mechanisms by which YHIEPV increases leptin sensitivity. SOCS-3, PTP1B, and TCPTP have been identified as key mediators that directly inhibit leptin signaling^25–31^. HFD intake has also been shown to promote the hypothalamic activation of multiple inflammatory and stress response pathways, such as proinflammatory cytokines (IL6, IL1β, and TNFα) and EPAC/Rap1 signaling, which decreased leptin responsiveness in the hypothalamus^7–11,32–35^. All of these factors are up-regulated in the hypothalamus by HFD-induced obesity.

**Figure 4:**
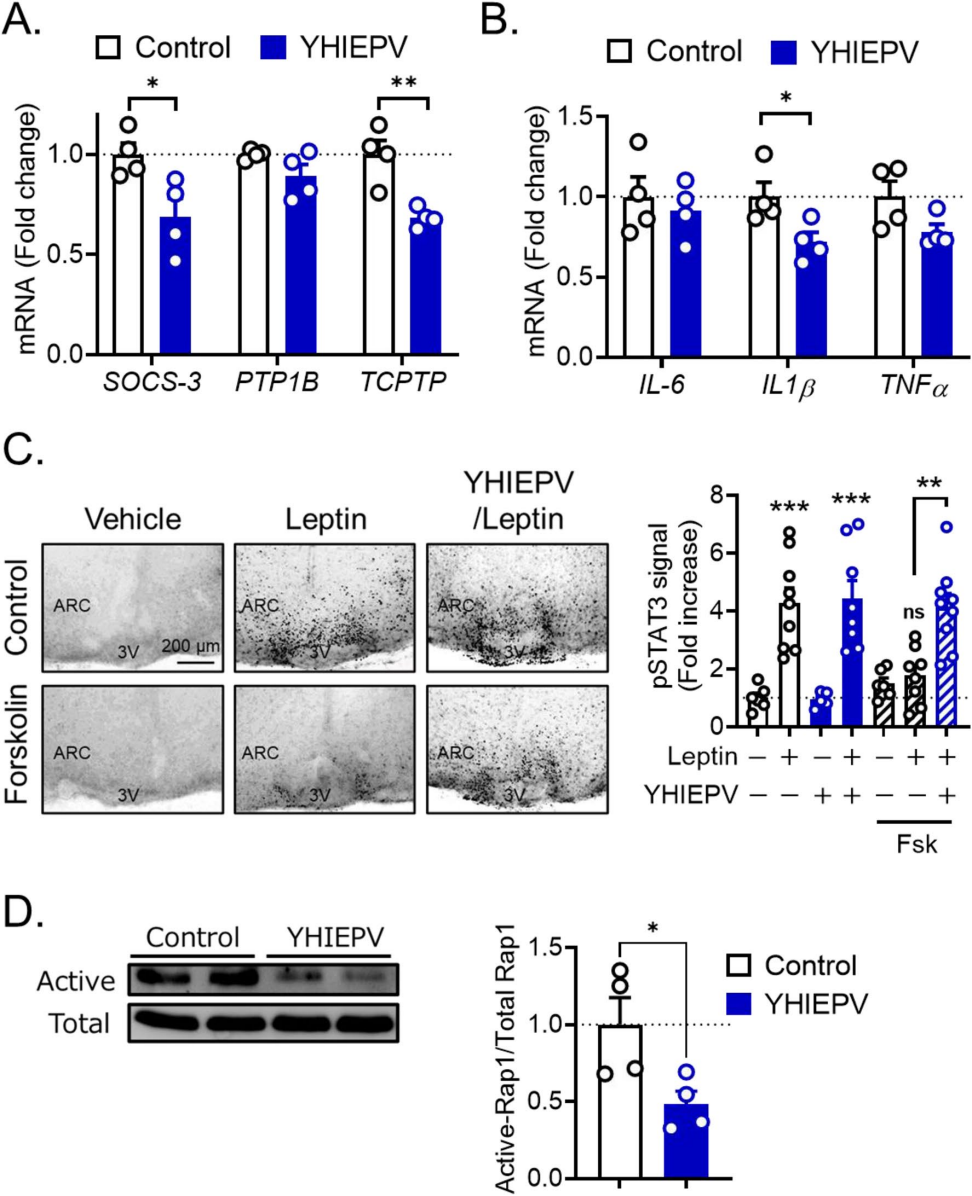
YHIEPV reduces central responses of proinflammatory-related factors and Rap1 activation. (**A**, **B**) Hypothalamic expression of genes involved in decreased leptin responsiveness in YHIEPV-treated and control C57BL/6 mice. The hypothalamus was collected from 4-month-old HFD-fed male mice after 5 days of the YHIEPV treatment (0.3 mg/kg, p.o.). YHIEPV reduced the hypothalamic mRNA expression of *Socs-3*, *Tcptp* (**A**), and *IL1β* (**B**). Mice were fasted for 3 h (from 8 to 11AM) before the collection of hypothalami. (**C**) Effects of YHIEPV on forskolin-induced cellular leptin resistance in organotypic hypothalamus slices. Slices were incubated with forskolin (Fsk, 20 μM) in the presence or absence of YHIEPV (100 μM) for 6 h and then stimulated with leptin (60 nM, 60 min). Leptin-induced pSTAT3 is shown (Left). Scale bar, 200 μm. Quantification of hypothalamic pSTAT3 (Right) (n = 5–9/group) in slices. (**D**) Western blot images (left) and quantification (right) of the amount of the active form of Rap1 in the brains of HFD-induced obese C57BL/6 mice treated with YHIEPV (0.3 mg/kg, p.o., for 5 days) or vehicle (n = 4/group). *p < 0.05, **p < 0.01, ***p < 0.001 for the *t*-test in (**A**), (**B**), and (**D**) or a one-way ANOVA followed by Tukey’s multiple comparison test in (**C**). All error bars are SEM.

Since YHIEPV increased in vivo leptin sensitivity in HFD-fed obese mice and protected against palmitic acid-induced in vitro cellular leptin resistance, we investigated whether YHIEPV affected the factors that negatively regulate leptin signaling in the hypothalamus. The oral administration of YHIEPV (0.3 mg/kg, 5 days, twice a day) significantly reduced the hypothalamic expression levels of *Socs-3*, *TCPTP*, and *IL1β* in obese mice (Fig. 4A,B). Under these conditions, YHIEPV also exhibited hypophagia without affecting body weight (Fig. S2). In addition, we observed that a single oral injection of YHIEPV showed anorexigenic effects in HFD-fed mice (Fig. S3). In agreement with anorectic responses, YHIEPV-induced HFD intake reduction was significantly blocked by the central administration of a melanocortin 4 (MC_4_) receptor antagonist. These findings suggest that, in addition to its role in enhanced leptin responsiveness, YHIEPV regulates food intake under hypercaloric feeding.

Consistent with previous findings, leptin-induced pSTAT3 levels were reduced by a treatment with forskolin, which activates Epac/Rap1 signaling (Fig. 4C), whereas leptin increased pSTAT3 levels in control mice (Fig. 4C). The pretreatment with YHIEPV abolished forskolin-induced cellular leptin resistance in slices (Fig. 4C). To further confirm the effects of YHIEPV on Rap1 signaling, we assessed the active (GTP-bound) form of Rap1 and found that YHIEPV reduced the level of GTP-bound (active) Rap1 in the brains of obese mice (Fig. 4D). This result suggested that Rap1 signaling in the CNS was involved in YHIEPV-induced increases in leptin responsiveness. Therefore, we revealed a previously unrecognized mechanistic relationship between the food-derived peptide and Rap1 signaling in hypothalamic leptin sensitivity.

## Discussion

An excess calorie intake, such as HFD, is associated with decreased leptin sensitivity. Leptin is a hormone that is secreted by adipocytes and mediates energy homeostasis by reducing food intake and body weight, and the CNS is involved in the biological responses of leptin resistance to obesogenic conditions. Therefore, it is interest to identify orally active compounds that increase leptin sensitivity in the CNS. Hormonal peptides, such as leptin, insulin, and GIP, are produced in the periphery (gastrointestinal tract and adipose tissue) and influence energy homeostasis in the brain^3–6,10,36–40^. Bioactive peptides are also produced in the gastrointestinal tract and provide additional health or medical benefits other than the usual nutritive value of food. The digestion of proteins in the gastrointestinal tract induces the release of numerous peptides, some of which have multiple physiological functions, such as improvements in glucose homeostasis, appetite regulation, cholesterol-lowering, anti-depressive-like, and anti-anxiety-like effects, improvements in cognitive function, and memory-enhancing activities^14,41,42^. Some bioactive peptides are also effective when orally administered in vivo^14,16,43–47^. Therefore, dietary proteins are recognized as a source of biologically active peptides, which have potential as orally active compounds to improve leptin sensitivity.

In the present study, we demonstrated a novel intervention to mitigate obesogenic conditions. Our ex vivo experiments revealed a previously unidentified nutritional link between plant-derived peptides and neuronal leptin signaling; YHIEPV, also named rALP-2^16^, derived from the protease digestion of the major green leaf protein Rubisco in the gastrointestinal tract, mimicking postprandial digestion, increased leptin sensitivity. These results were supported by those obtained in in vivo experiments. Mice orally administered YHIEPV did not develop resistance to the anorectic and cellular effects of exogenous leptin under HFD conditions. The YHIEPV treatment was associated with the long-term maintenance of weight loss even after the cessation of the leptin treatment. While saline-control mice only showed leptin-induced pSTAT3 in the ARC, YHIEPV increased leptin-induced pSTAT3 levels not only in the ARC, but also in the ventromedial nucleus (VMH). The molecular mechanisms mediating this effect are unclear, but may involve leptin signaling in the VMH because the selective deletion of leptin receptors in VMH SF1 neurons has been shown to reduce body weight gain^48^. These results prompted us to examine potential interactions between YHIEPV and the control of the whole-body energy balance.

Body weight gain and cumulative food intake were significantly lower in HFD-induced obese mice orally administered YHIEPV daily than in saline-control mice. In addition to its oral effects, the central administration of YHIEPV markedly reduced body weight gain under HFD. YHIEPV-induced anti-obesity effects were more robust after its central administration than after its oral administration, suggesting that YHIEPV directly interacts with the brain. Within hypothalamic nuclei, the ARC, which densely expresses leptin receptors and is adjacent to the third ventricle, has a weak and incomplete blood–brain barrier (BBB), which potentially allows the access of peripherally derived circulating bioactive peptides, nutrients, and gut hormones via the bloodstream^49,50^. Further studies are needed to establish whether YHIEPV directly enters and acts on the brain to enhance neuronal leptin responsiveness. Alternatively, YHIEPV may act on targets in the gastrointestinal tract, and peptide signals may then be transferred from the periphery to the brain via a neural or humoral pathway.

To investigate the effects of YHIEPV on in vivo neuronal leptin responsiveness, we used two different mouse strains, ddY and C57BL/6. Although C57BL/6 mice are more widely used as dietary obese mice, ddY mice also develop marked hypertriglyceridemia in response to HFD^51^. ddY mice were fed HFD only for 5 weeks and their body weight increased to approximately 50 g, as shown in Fig. 3A. Even in the short-term HFD challenge with ddY mice, leptin responsiveness decreased, as shown in Fig. 2. Under these conditions, YHIEPV appeared to enhance the anorectic response of centrally administered leptin and the daily oral administration of YHIEPV protected against further body weight gain. On the other hand, C57BL/6 mice were used in experiments on the central administration of YHIEPV and in the hypothalamic signaling analysis because organotypic hypothalamus slices were obtained from C57BL/6 pups and the treatment of hypothalamic slices with YHIEPV enhanced leptin responsiveness. Based on these results, we demonstrated that the YHIEPV treatment enhanced leptin responsiveness in ddY and C57BL/6 mice. Furthermore, to identify the hypothalamic signaling molecules potentially affected by the oral administration of YHIEPV, YHIEPV was only administered to mice for five days and significant differences were observed in food intake without changes in the body weight of C57BL/6 mice, suggesting that the primary effect of YHIEPV was hypophagia. YHIEPV showed similar phenotypes in ddY and C57BL/6 mice, as shown in Fig. S2.

We then investigated the mechanisms by which YHIEPV increased leptin sensitivity. Inflammatory and stress response pathways, such as the hypothalamic induction of SOCS-3, inflammation, and EPAC/Rap1 signaling, are known to actively respond to HFD and decrease leptin responsiveness. Based on the results of the qPCR analysis, hypothalamic *SOCS-3* and inflammatory *IL1β* were markedly lower in YHIEPV-treated HFD-fed obese mice than in body weight-matched control mice. This result suggested that YHIEPV prevented HFD-induced increases in SOCS-3 and IL1β in the hypothalamus to increase leptin responsiveness. Furthermore, we previously demonstrated that the genetic deletion of Rap1 in forebrain neurons protected mice against HFD-induced obesity and decreased leptin responsiveness. The pharmacological inhibition of brain-Rap1 signaling by the central administration of the Epac2 inhibitor ESI-05 also restored the leptin-induced suppression of food intake, reductions in body weight, and the phosphorylation of STAT3. The daily central infusion of ESI-05 significantly reduced body weight and food intake in HFD-induced obese mice. Therefore, Epac-Rap1 signaling has potential as a target for anti-obesity effects^8^. To investigate whether Epac-Rap1 signaling is involved in YHIEPV-induced increases in leptin sensitivity, we employed a powerful unique in vitro system using ex vivo organotypic hypothalamus slices, which enabled us to manipulate signaling cascades within the hypothalamus. Forskolin activates Epac-Rap1 signaling and causes cellular leptin resistance. The pretreatment with YHIEPV inhibited cellular leptin resistance induced by forskolin. Moreover, the oral administration of YHIEPV reduced the level of the GTP-bound active form of Rap1 in the brain, confirming our ex vivo results. Since brain Rap1 activation has emerged as a causative factor for decreases in leptin responsiveness and obesity, the reduced activity of brain Rap1 signaling may coordinately contribute to the phenotypes of the oral YHIEPV treatment. We herein demonstrated that the oral administration of the bioactive peptide enhanced neuronal leptin responsiveness and significantly reduced Rap1 activity in the brain.

The mechanisms linking peripherally derived bioactive compounds to neuronal leptin responsiveness and obesity are being intensively investigated. Since the Epac-Rap1 pathway may be activated by GPCRs via Gs-cAMP signaling^10,52^, we hypothesized that a peripherally derived Gi-GPCR ligand that inhibits cAMP signaling may enhance cellular leptin responsiveness. Through a combination of pharmacological, in vivo mouse model, and ex vivo brain explant studies, we demonstrated that the green leaf Rubisco-derived bioactive peptide YHIEPV acted on the hypothalamic metabolic circuitry to increase leptin responsiveness and protect against dietary obesity by inhibiting Epac-Rap1 signaling in the brain. Collectively, the present results suggested that YHIEPV activated Gi-GPCR signaling. YHIEPV was previously shown to suppress the increases induced in intracellular cAMP levels by forskolin in a dose-dependent manner in Neuro-2a cells^16^. In further support of this hypothesis, bioactive peptides derived from food proteins have been identified as GPCR ligands^40,53^. We also found several bioactive peptides derived from animal and plant proteins that have affinities for GPCRs^14^. As shown in Fig. S3, YHIEPV-induced reductions in food intake were inhibited by the central administration of a MC_4_ receptor antagonist. Since MC_4_ receptor signaling is a crucial mediator of leptin-induced reductions in food intake and body weight, this result suggests that the oral treatment with YHIEPV activates the central melanocortin system. Further studies are needed to identify the target GPCR for YHIEPV to exert its effects via the gut-brain axis, and whether orally administered YHIEPV crosses the BBB.

In conclusion, the present study demonstrated the potential of the plant Rubisco-derived peptide YHIEPV as a leptin sensitizer, and revealed a previously unidentified link between exogenous food-derived compounds and central Rap1 signaling. This is the first study to report a food-derived bioactive peptide that increases central leptin sensitivity.

## Supplementary Information

Supplementary Figures.

## Data Availability

The datasets used and/or analyzed during the present study are available from the corresponding author upon reasonable request.
