# Supplementary material for: An orally active plant Rubisco-derived peptide increases neuronal leptin responsiveness
Source: Sci Rep. 2022 May 21;12:8599. doi: 10.1038/s41598-022-12595-6 (PMC9124197; doi:10.1038/s41598-022-12595-6)

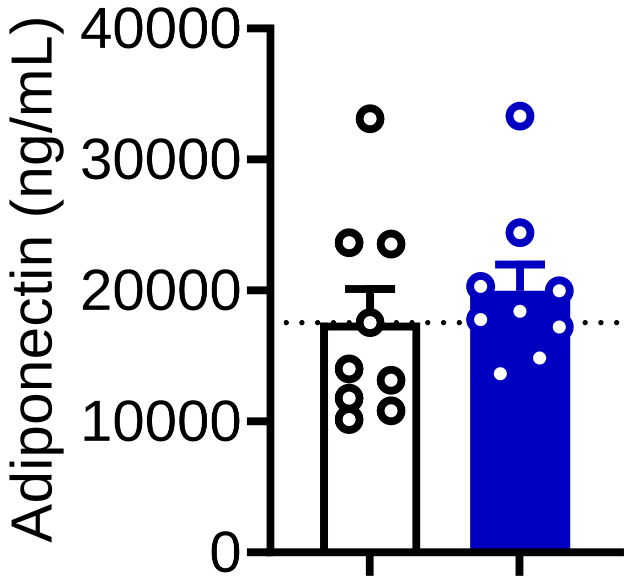

Figure S1. Effects of YHIEPV on adiponectin secretion

HFD-fed obese ddY mice (HFD for 2 months, n =9/group) received oral YHIEPV (0.3 mg/kg for 35 days) once a day. Plasma adiponectin levels were measured on day 35. Error bars are the SEM.

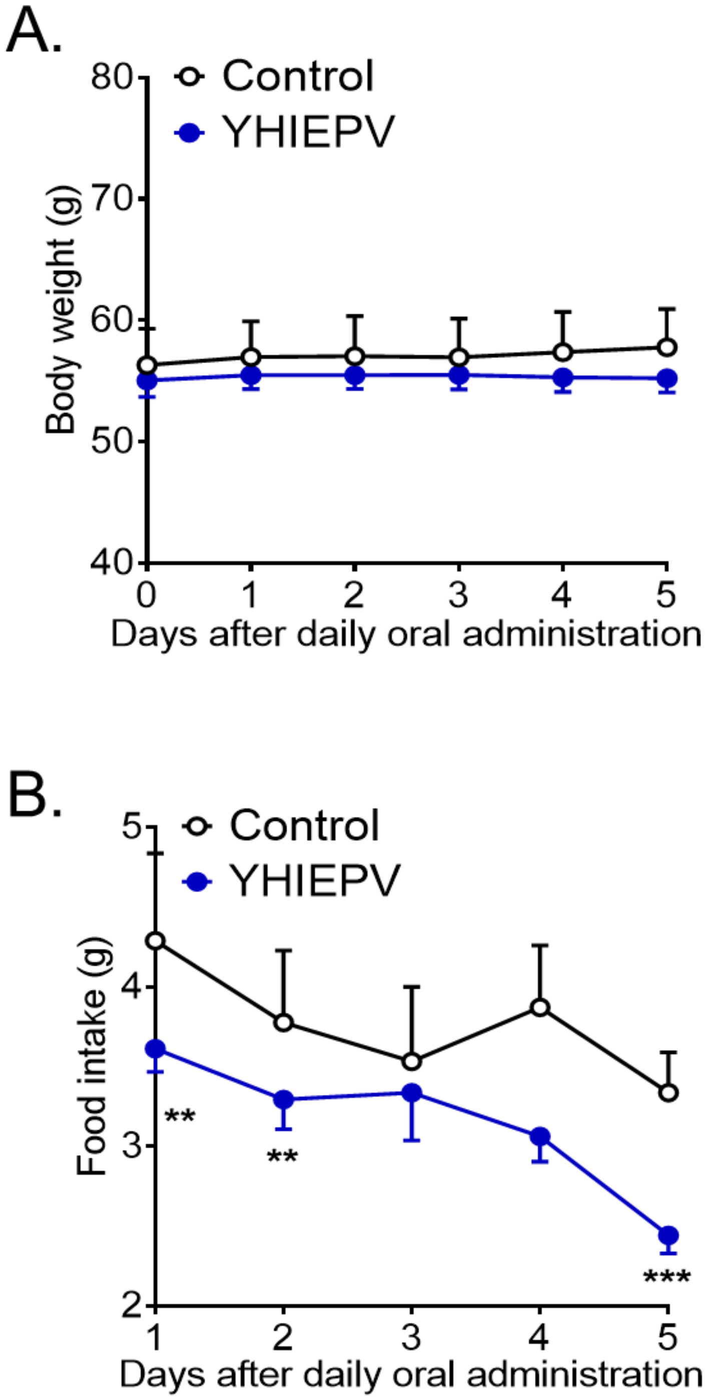

Figure S2. Orally administered YHIEPV decreased food intake in C57BL/6 mice

Four months HFD-fed C57BL/6 mice were received YHIEPV (0.3 mg/kg, p.o.) or vehicle treatment for 5 continuous days (n = 4/group). Shown are body weight changes (A) and daily food intake (B). $^{**}p < 0.01$, $^{***}p < 0.001$ for two-way ANOVA followed by Bonferroni's multiple comparisons tests in (B). All error bars are the SEM.

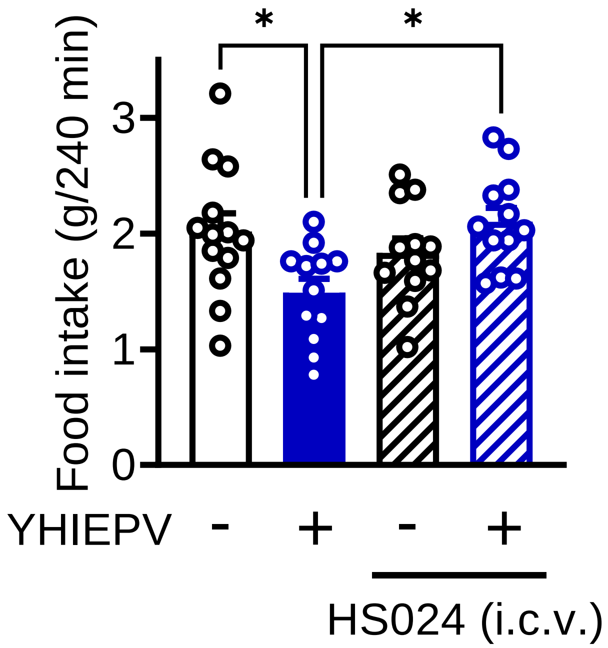

Figure S3. Orally administered YHIEPV decreased high-fat diet intake via central melanocortin system

Five weeks HFD-fed ddY mice were received YHIEPV (0.3 mg/kg, p.o.) or vehicle treatment, followed by HS024 (0.1 nmol/mouse, i.c.v.). Food intake were measured after 4 hours of treatment.

*$p < 0.05$ for one-way ANOVA followed by Tukey’s multiple comparison test. All error bars are the SEM.

Full unedited gels for Figure 4D

A. GTP-bound Active Rap1

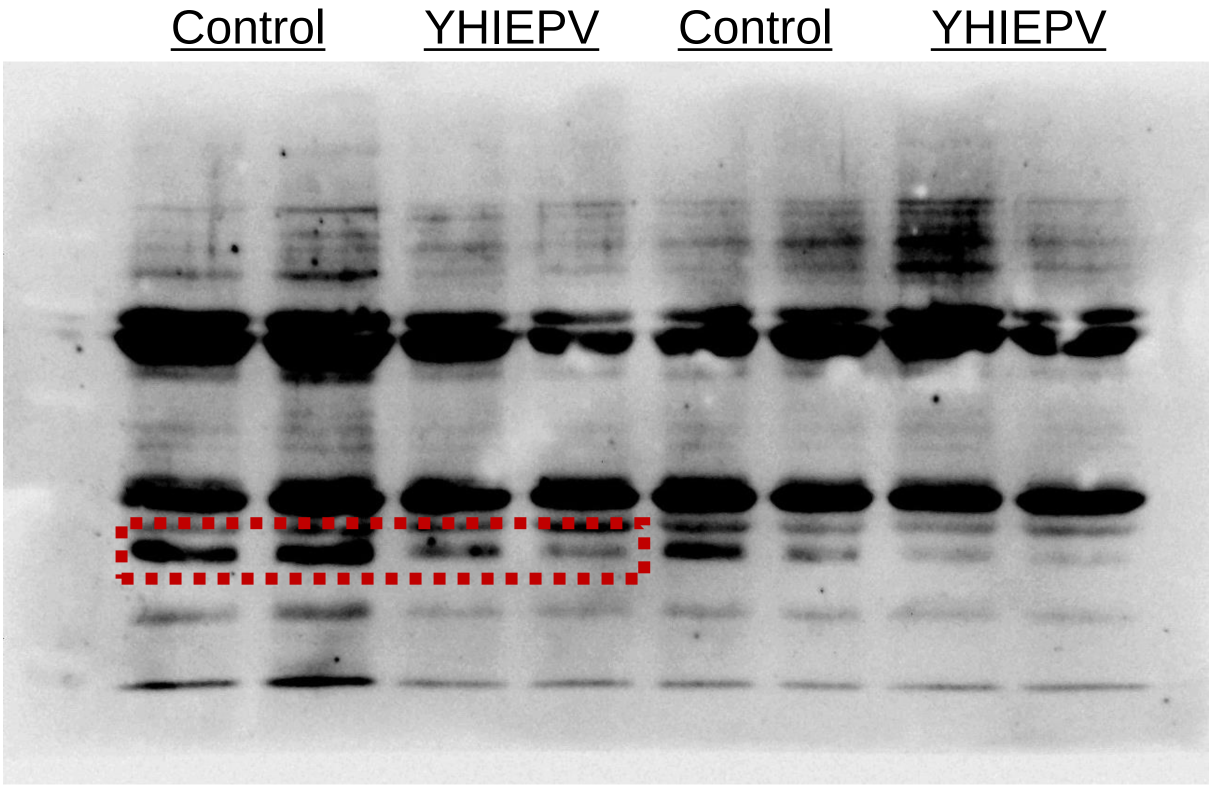

B. Total Rap1

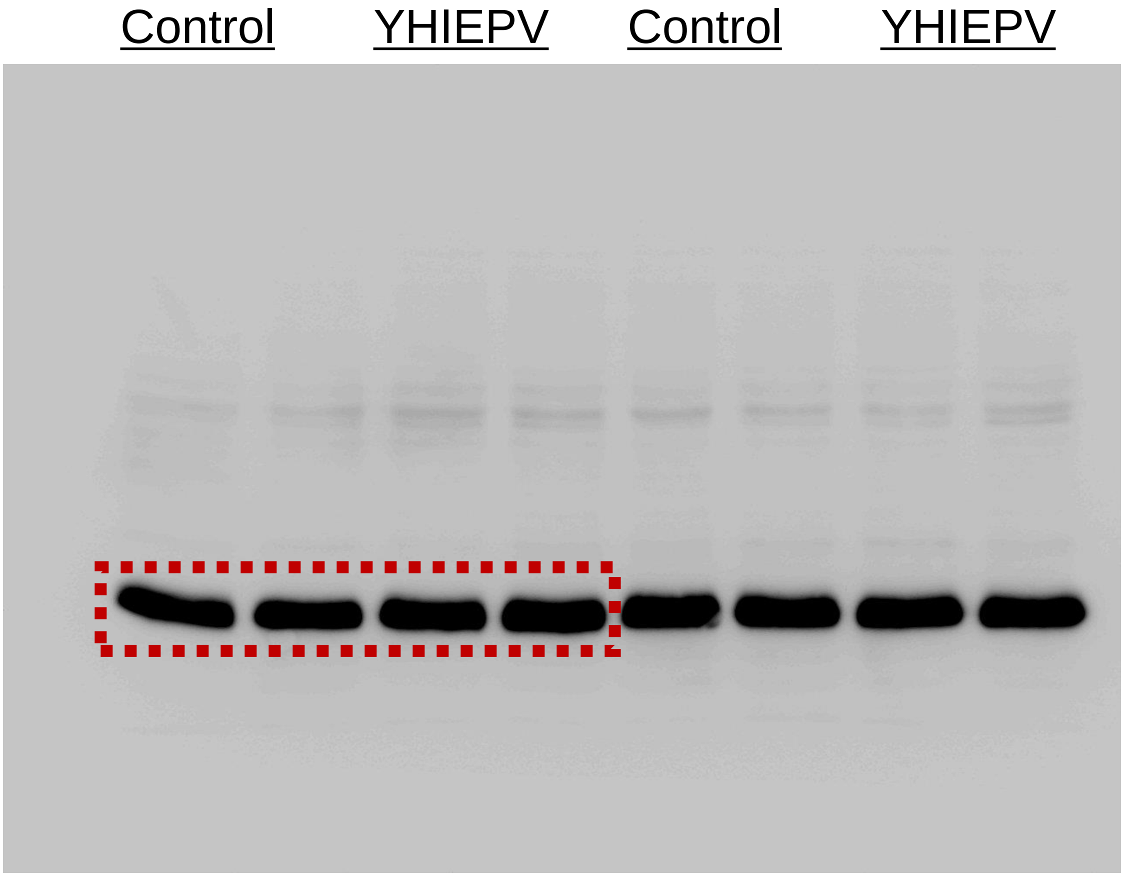

Supplement: Supplementary file 1 — Supplementary Figures. [file 41598_2022_12595_MOESM1_ESM.pdf]
